# Characterization of toxin systems of Paenibacillus strains isolated from honeybees

**DOI:** 10.1038/s41598-025-12956-x

**Published:** 2025-08-26

**Authors:** Anthony Pannullo, Ephantus J. Muturi, Christopher A. Dunlap

**Affiliations:** Crop Bioprotection Research Unit, Agricultural Research Service, Department of Agriculture, National Center for Agricultural Utilization Research, Peoria, IL USA

**Keywords:** Agroecology, Evolution, Applied microbiology, Microbial genetics, Pathogens

## Abstract

*Paenibacillus* is a large genus of Gram-positive bacteria isolated from a wide range of sources. Members of this genus have attracted significant research as plant growth promoters as well as pathogens of both plants and animals. Extensive studies have been conducted on *Paenibacillus larvae*, the causative agent of American Foul Brood in honeybees, but little is known about other closely related species associated with honeybees, such as *Paenibacillus apiarius* or *Paenibacillus thiaminolyticus*. These bacterial species have been isolated from dead honeybee larvae and are believed to be secondary invaders as opposed to primary pathogens. We utilized genome mining efforts to explore the number and diversity of toxins present in multiple species of *Paenibacillus*, with major emphasis on a potentially entomopathogenic clade that includes *Paenibacillus alvei*, *P. apiarius*, and *P. thiaminolyticus*. Our results show that strains of these species contain a variety of potentially insecticidal toxins, including highly conserved Spp-like and Mpp-like toxins. These toxins are absent in *P. larvae*. We also found several ADP-ribosylases which were shared between *P. larvae* and several strains from this entomopathogenic clade. Tc toxins, a class of insecticidal toxin not often found in Gram-positive bacteria were found in in *P. apiarius* and *P. alvei*. These findings suggest the potential for *Paenibacillus* species in this clade to be harnessed as sources of host specific and pollinator-friendly biopesticides.

**Supplementary Information:**

The online version contains supplementary material available at 10.1038/s41598-025-12956-x.

## Introduction

*Paenibacillus* is a large genus of Gram-positive, spore forming bacteria found frequently in soil, plant, and insect associated environments^1^. Some *Paenibacillus* species have been studied and utilized for their plant growth promotion capabilities. For example, *Paenibacillus polymyxa*, which exists in the rhizosphere promotes plant growth through production of antimicrobials, nitrogen fixation, and phosphate solubilization^2–5^. However, some *Paenibacillus* species are known to be entomopathogenic. The best characterized example of insect pathogen from this genus is *Paenibacillus larvae*, the causative agent of American Foulbrood (AFB) in honeybees^6–9^. Infection of the honeybee colony with *P. larvae* typically occurs when the worker bees feed the larvae with food from contaminated sources. Other sources of infection include contaminated bees from a nearby colony and contaminated bee keeping equipment. AFB is the most destructive bacterial disease of the honeybee larvae, causing death within 4–12 days depending on the strain^6,7^. AFB is extremely difficult to control due to widespread resistance of *P. larvae* to antibiotics and the highly persistent nature of *P. larvae* spores. Thus, incineration of infected hives is the most viable option for combating AFB outbreak and causes substantial economic losses to producers^1,6–8^.

The mechanisms of pathogenesis in *P. larvae* are not necessarily consistent or conserved among all strains. *P. larvae* strains are typed according to their specific genotype, referred to as enterobacterial repetitive intergenic consensus (ERIC). Currently, there are 5 ERIC types (I, II, III, IV, and V) with ERIC I and II being the most commonly isolated genotypes from infected colonies^8–11^. Pathogenesis of ERIC I genotype is primarily mediated by a toxin system made up of two proteins, Plx1 and Plx2. This toxin system causes significant damage to the gut epithelium of honeybee larvae once ingested *P. larvae* spores germinate in the larval gut^12^. In contrast, ERIC II genotype is characterized by the lack of toxin genes or the presence of nonsense mutations within toxin encoding genes. Instead, ERIC II strains rely on production of a proteinaceous S-layer as a component of the cell envelope. This S-layer allows the vegetative cells to better adhere to epithelial cells in the gut and persist long enough to establish an infection^13^. There are several known protein families of insecticidal toxins. These toxins are generally distinguished based upon their structure, and recent efforts to normalize the nomenclature of the toxin families has facilitated toxin research. It is well known that toxigenic strains of *P. larvae* function via MTX1 toxins. Many strains of *P. larvae* have also been shown to harbor binary toxin systems (Vpa/b type genes)^6,12^. *P. alvei* is known for its production of alveolysin, a sphaericolysin homolog, and *P. popilliae* is known for its production of Cry proteins^14^.

*P. larvae* is regarded as the only true honeybee pathogen among the *Paenibacillus* species. However, there are other *Paenibacillus* species that cause infection in other insects. *Paenibacillus popilliae* and *Paenibacillus lentimorbus* infect larvae of Japanese beetles (*Popillia japonica*) causing the milky spot disease^14,15^. Cry proteins have been identified in both species and are thought to play a major role in beetle mortality^14,15^. Cry proteins represent an insecticidal toxin class that have been extensively studied due to their prolific production in the well-studied insecticidal bacteria, *Bacillus thuringiensis*^16^. Cry proteins function by causing perforations within the epithelium of the insect gut, resulting in death of the insect. Despite the conserved mechanism, different Cry proteins have different insecticidal capabilities among insect species^17–20^. Cry proteins selectively target insect gut epithelial cells via binding of receptors on epithelial cell surfaces after the cry proteins have been processed by extracellular proteases. This receptor binding activity is what confers insect specificity to the activity of cry proteins^21,22^.

Another species, *Paenibacillus alvei*, is often isolated from honeybee hives infected with European Foulbrood (EF), caused by *Melissococcus plutonius*^23–25^. Although this bacterium has been shown to infect bee larvae under laboratory conditions, it is not clear whether *P. alvei* is a major contributor to EF pathogenesis. *P. alvei* produces a thiol-activated cytolysin, alveolysin^26^ which is homologous to several toxins that are implicated with human disease including listeriolysin O, perfringolysin O, and streptolysin O^26–28^. *P. alvei* is not a commonly isolated human pathogen and alveolysin does not appear to target mammalian cells despite having a similar mechanism of action to the previously listed toxins. This class of cytolysins function by recognizing and binding to cholesterol found within eukaryotic membranes. This cholesterol binding leads to oligomerization of toxin proteins within the membrane, eventually forming numerous pores throughout the membrane and resulting in cell lysis^26,27^.

Alveolysin is also homologous to the studied insecticidal protein, sphaericolysin, produced by *Lysinibacillus sphaericus*. It is known that sphaericolysin contributes to insecticidal potential in *L. sphaericus* and thus this class of toxin proteins are referred to as Sphaericolysin-like-proteins (Spp)^29^. Additionally, *L. sphaericus* is known to produce ETX/MTX-2 (Mpp) toxins which also function as pore forming proteins to disrupt eukaryotic cell membrane structure, resulting in lysis^30,31^. These toxins have been noted to play a role in mosquitocidal activity present in *L. sphaericus*, and genome mining has revealed that certain strains of *P. larvae* are predicted to contain MTX2 homologs, though it is not established if these toxins play a role in honeybee pathogenesis^6,32^. *Paenibacillus dendritiformis* and more recently *Paenibacillus melissococcoides* have also been isolated from honeybee hives infected with EF^25^, but their role in disease pathogenesis remains poorly understood. There is also preliminary evidence of occurrence of *Paenibacillus apiarius* in hives infected with American Foulbrood, but its role on disease progression is not clear^33,34^. But the presence of these strains of *Paenibacillus* in dead bee larvae, suggest that may have some entomopathogenic activity. In addition, these strains are part of a phylogenetic clade that contains known entomopathogens, which suggests this may represent a clade of entomopathogens arising from the same evolutionary past.

Most studies on insecticidal potential of *Paenibacillus* species have mainly focused on P. *larvae* due to its ability to devastate honeybee hives. However, other species of *Paenibacillus* should be investigated as potential factors in honeybee pathogenesis, and as pathogens of other insects. These studies could facilitate the identification of new *Paenibacillus* strains, or toxins with potential biocontrol applications, and provide insights into how these toxins have evolved within the genus. This study investigated sequence diversity among toxins in *Paenibacillus* strains to develop a better understanding of the evolutionary history of toxin development in these strains. Our objective was to dissect the evolutionary relationship between the various toxins and the strains they existed in. To accomplish this, we compared newly sequenced genomes of *P. larvae*,* P. apiarius*,* P. thiaminolyticus*, and *P. alvei* strains with existing genomes deposited in NCBI. We then performed comparative genomic analysis including phylogenetic tree construction, toxin identification, and whole-genome alignments to understand the presence and conservation of toxins within these species and strains. We found that many of the *Paenibacillus* strains that are predicted to be toxigenic based on genome annotation, exist within this same clade and that this, newly identified, putative entomopathogenic clade is evolutionary distinct from *P. larvae*. Our findings suggest that this clade has evolved to consist of opportunistic insect pathogens through the dispersal of conserved insecticidal proteins. This clade should be extensively studied for impact on honeybee pathogenesis and for potential application as biopesticides.

## Materials and methods

### DNA isolation and genome sequencing

The 50 sequenced strains were grown to the exponential growth phase in 4 ml of Tryptone glucose yeast extract media for 24 h at 200 rpm and 37^◦^C. DNA extraction was performed using the SDS-proteinase K method^35^. The sequencing libraries were prepared using Illumina DNA prep kit following the manufacturer’s protocol. The libraries were sequenced using a Novaseq X sequencer using 2 × 150 bp paired-end sequencing. The resultant sequencing reads were quality trimmed to an error probability of 0.05 using CLC genomics workbench v24.01. A *de novo* assembly was performed using default parameters with CLC genomics workbench v24.01. Genome-based taxonomy was determined using the type strain genome server^36^ and listed in Table S1.

### Acquisition of FASTA files

FASTA files for all genomes used in this study can be found on NCBI SRA under the Bioproject number PRJNA805124. Contigs for genomes were annotated using Prokka (v1.14.5) with default parameters^37^. Fifty strains of *Paenibacillus* were sequenced in this study, additional genomes were downloaded from NCBI, the accession number and citation (if available) for each genome can be found in Table S1.

### Phylogenetic tree construction

Maximum-likelihood phylogenetic trees were constructed using the Validated Bacterial Core Genes (VBCG) program (v1.3)^38^. Nucleic Acid FASTA files for each of the 117 strains displayed were utilized as the input for the program. VBCG was run with default parameters. The tree was plotted using the ggplot2 (v 3.5.1) and ggtree (v1.14.6) packages available in R (v 4.3.0).

### Genome mining of bacterial pesticidal proteins

The ~ 1500 curated sequences available from the Bacterial Pesticidal Protein Database (BPPD)^39^ were downloaded on 1 November 2024 and used to construct a custom BLAST available for the stand-alone distribution of BLASTp (v 2.12). This was done to allow a more targeted BLAST approach to searching for these pesticidal proteins. The BLAST search for each genome was performed by using a standard BLOSUM62 matrix and was filtered based on an *e-*value threshold < = 1e^−20^, a bit score threshold of > = 300, and a percent identity threshold > = 35%. If there were multiple BLAST hits for any given protein, the hit with the lowest *e*-value and highest bit-score was selected to ensure that each protein had a singular prediction. Identified toxins were cross validated using a second toxin identification method utilizing the BtToxin_Digger (v1.0.2) with default parameters^40^. Since the BtToxin_Digger currently utilizes some of the older toxin naming schema, we chose to present the data derived from the BLAST search of the BPPD which utilizes the modernized nomenclature. However, the confirming BtToxin_Digger data can be found in Table S2. The counts of protein families were then tabulated for toxin-containing strains and displayed in Fig. 2.

### Principle component analysis

Principal component analysis was used to assess whether identified toxins clustered more closely based on protein family or the species in which they were identified. Positionally weighted matrix was constructed for each toxin amino acid sequence and then used as the input in the ‘prcomp’ function in base R with default parameters. The number of clusters were selected by validating through a scree plot (Figure S1).

### Mauve alignments

To visualize genomic neighborhoods of conserved toxin genes, whole genome alignments were performed using MAUVE (v2.4.0)^41^ using the progressiveAlignment function with default parameters. Due to the intense computation requirements for genome alignment, a subset of selected strains was used to construct the alignments, rather than all species or all strains for a given species. Strains were selected on the basis that they contained the common toxins for that species. Alignment images were then directly exported from the mauve software.

### Additional toxin identification

BPPD and BtToxin_Digger genome mining tools exclusively look for established pesticidal toxins from the commonly found protein families, such as Cry, Mtx, Mpp, ect. However, we wanted to understand the total toxin profile for each of these strains, including any toxins that may be otherwise missed from these analyses. To do this, we acquired 2 million putative toxin sequences from the Toxinome database on 2 November 2024^42^ and constructed a phmmer hmm database (v3.4)^43^ using the Toxinome sequences as inputs. The acquired sequences included many ATP-binding cassettes that are associated with or are predicted to be associated with either toxin activation/production or resistance to certain antimicrobial peptides. Due to the high degree of homology found within these proteins, and the relatively little information that is provided by identifying an ATP-binding cassette, we removed approximately 14,000 sequences from the list of 2 million sequences that had dedicated ATP-binding cassette proteins. To construct the hmm database, the sequences were first clustered into groups using CD-HIT (v4.8.1)^44^ with a similarity threshold of 0.6. This threshold was arbitrarily chosen to limit the number of clusters in subsequent steps, while maintaining sufficient homology. Each CD-HIT cluster was then separately aligned using MAFFT (v7.463)^45^ with default parameters. Alignments were constructed on a per-cluster basis rather than all sequences, due to the vast variety of proteins used. A single alignment of all proteins would be losing a lot of potentially important sequence and domain information. Each individual cluster was constructed into its own hmm profile, and all profiles were then merged into a singular hmm database. Using phmmer, this database was used to search through each of the 117 genomes for the presence of additional toxins or proteins that contain toxin-related domains.

### Toxin domain identification and similarity analysis

Conserved domains of TcABC toxins were identified using InterProScan (v102.0) on individual toxin sequences, searching against all available databases with default parameters^46^.

## Results

### The putative entomopathogenic clade of *Paenibacillus*

We were initially interested in identifying the evolutionary relationships between several *Paenibacillus* species that had been found isolated from dead honeybee larvae. These strains included *P. alvei*, *P. apiarius*, *P. thiaminolyticus*, and P. *larvae*. Of these species, only *P. larvae* is known to be actively pathogenic towards honeybees. Some strains of *P. alvei* have been tested for pathogenicity in honeybees, and can cause larval infection in certain laboratory circumstances, though not nearly to the degree of *P. larvae.* However, the question remains whether they are involved in pathogenesis as a secondary factor alongside *P. larvae* infection, and whether this potential pathogenesis is a shared trait amongst this clade of *Paenibacillus. *To examine the evolutionary relationship between these strains, we utilized the newly sequenced genomes presented in this study, along with several established genomes already available on NCBI to allow us to evaluate how these new sequences fit into the greater context of the *Paenibacillus* genus. Strain and genome information can be found in Table S1. The *Paenibacillus *genus has been represented in several phylogenetic trees, however, in this instance, we were particularly interested in the taxonomic distribution of these, newly sequenced strains (Table S1). We constructed a maximum-likelihood tree using VBCG and found that our phylogenetic tree strongly resembled the ones currently available. However, with the newly sequenced strains we were able to expand the representation of *P. alvei*, *P. apiarius*, *P. thiaminolyticus*, and *P. larvae*.

**Fig. 1:**
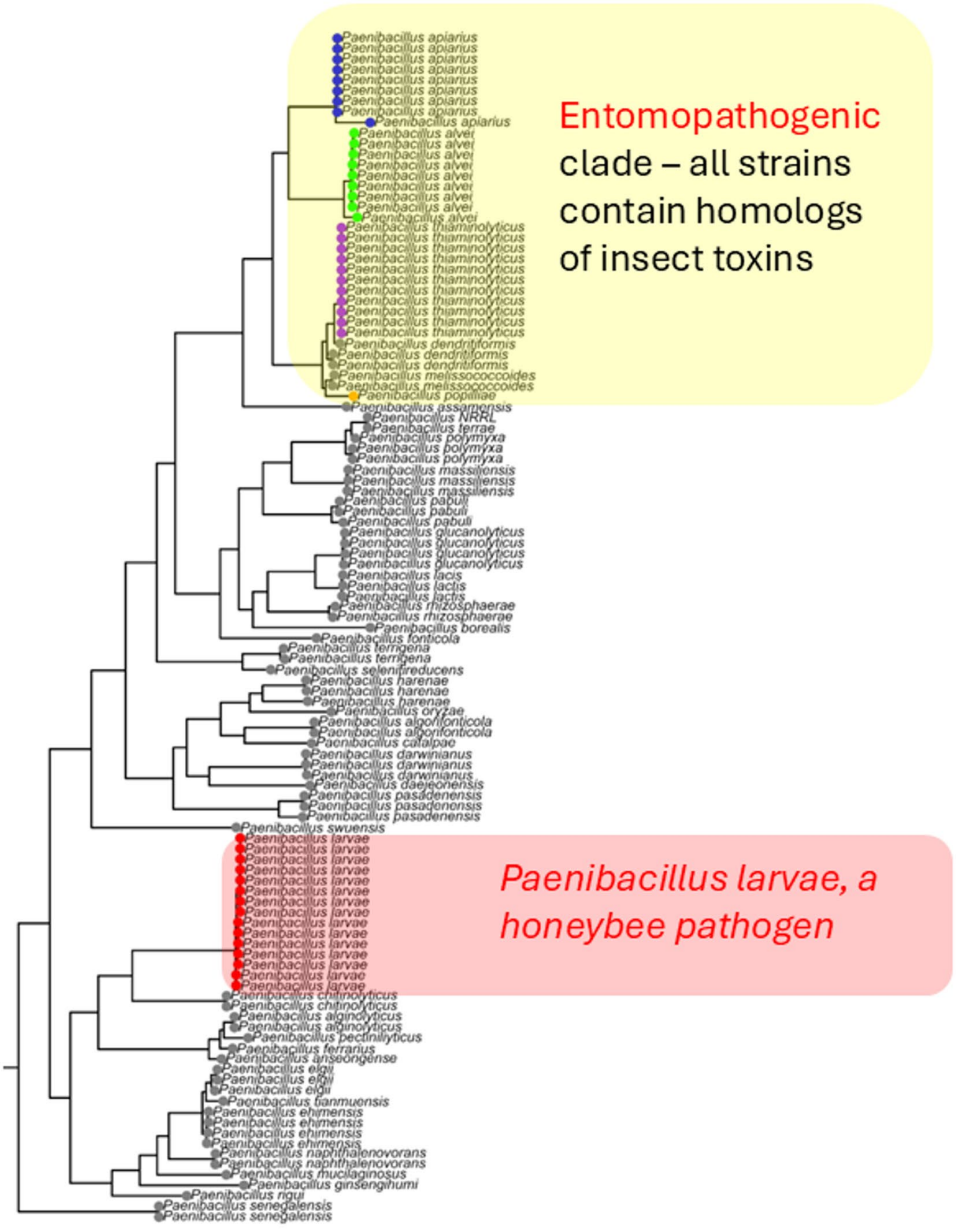
Phylogenetic Tree of Select *Paenibacillus* Species Reveals Potential Clade of Opportunistic Insect Pathogens. Maximum-likelihood tree was constructed using 117 *Paenibacillus* genomes spanning 42 species. The tree was constructed using VBCG (20 conserved proteins) run with default parameters and plotted using the ggtree library available in R. Species of potential pathogenic interest are highlighted in their own color. *Brevibacillus forte* was used as an outgroup and removed to conserve space.

From the phylogenetic tree (Fig. 1) we can see that *P. larvae* is quite separate from the rest of the sequenced strains, which otherwise cluster together in a singular clade (highlighted in Fig. 1). The presence of multiple clades of known insect pathogensindicates that insect pathogenesis has evolved multiple times within the *Paenibacillus* genus.

### Entomopathogenic clade harbors a variety of insecticidal toxins notably distinct from *P. larvae*

With the newly acquired genomes and strains, we were interested in determining how potential insecticidal toxins are distributed amongst this clade. Specifically, we were interested in evaluating whether the presence of insecticidal toxin encoding genes was unique to *P. popilliae* and *P. alvei*, or a common occurrence among related clades.

**Fig. 2:**
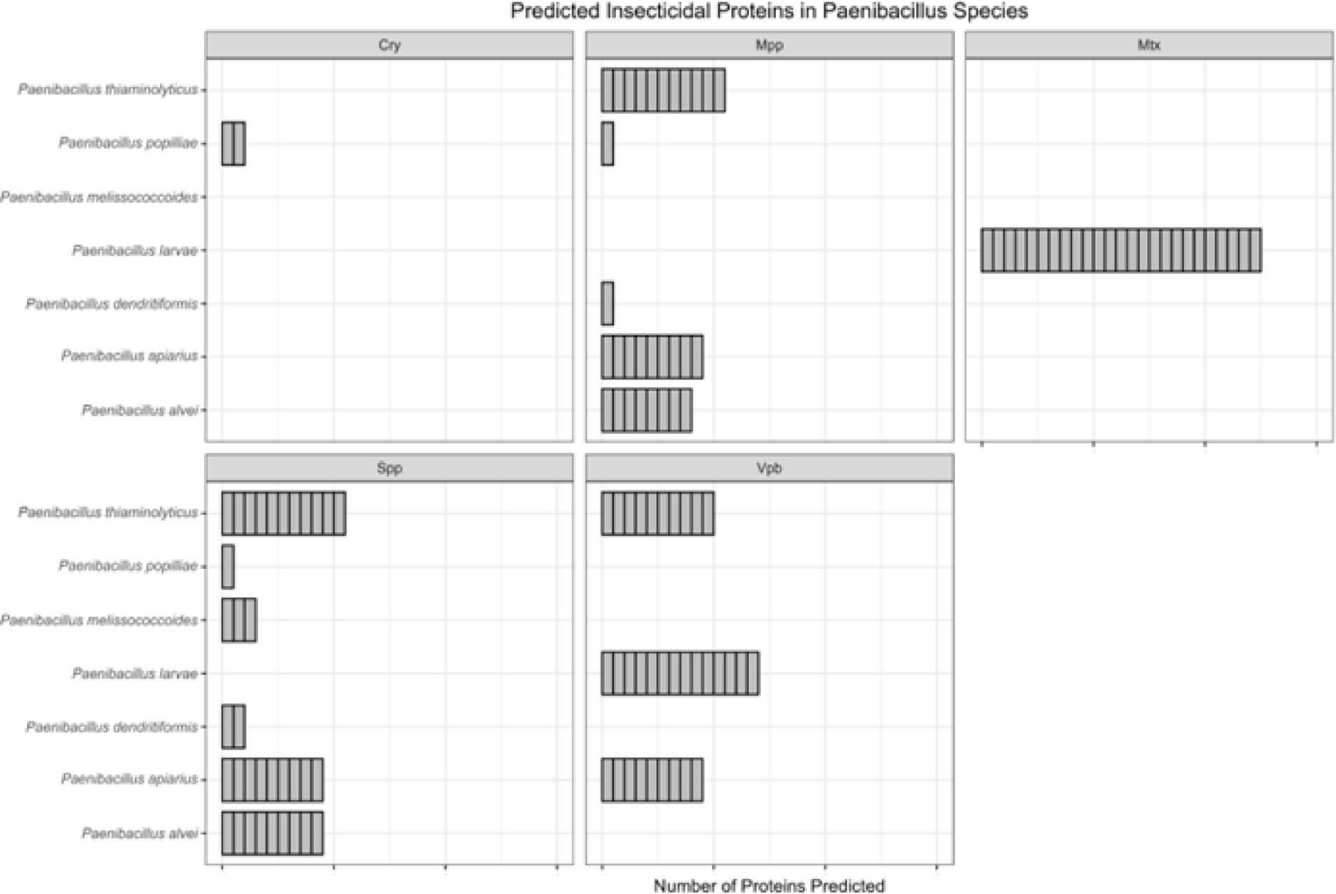
Putative Insecticidal Toxins Identified in 13 *Paenibacillus* Species. 117 *Paenibacillus* genomes were blasted against the Insecticidal Toxin Database containing 1427 curated insecticidal toxin protein sequences. Blast cutoffs were defined as follows: Identity Threshold > 40%, e-value Threshold < 1e-^10^, bit-score Threshold > 50. Protein sequences that met these parameters are included in the figure. Each individual rectangle (denoted by black border lines) represents an individual strain; the size of the rectangle indicates the number of toxins identified in that single strain.

To investigate this, we examined our set of sequenced genomes and NCBI-acquired genomes (Table S1) for the presence of insecticidal toxins. We found that five families of insecticidal toxins were represented among the 117 strains that were analyzed. These included Cry, Mpp, Mtx, Spp, and Vpb (Fig. 2). As predicted, only *P. larvae* appeared to harbor genes encoding MTX1-type toxins and *P. popilliae* was the only species that had evidence of Cry proteins. The most interesting results was the representation of Spp (Sphaericolysin-like) and Mpp (MTX2-like) type toxins among the putative entomopathogen clade. Many species in this clade have at least one representative of either Mpp or Spp type toxin, with some species, such as *P. alvei*, *P. apiarius*, and *P. thiaminolyticus*, having both toxin types. The Spp toxin type had the greatest taxonomic distribution, with some species existing outside our clade of interest harboring the toxins. Additionally, *P. thiaminolyticus*,* P. apiarius* and *P. larvae* possess both Vpa and Vpb proteins, components of binary toxin systems, with most containing two distinct Vpb proteins that are located next to each other in the genomes.

### Mpp and spp toxins are highly conserved among the entomopathogen clade

**Fig. 3:**
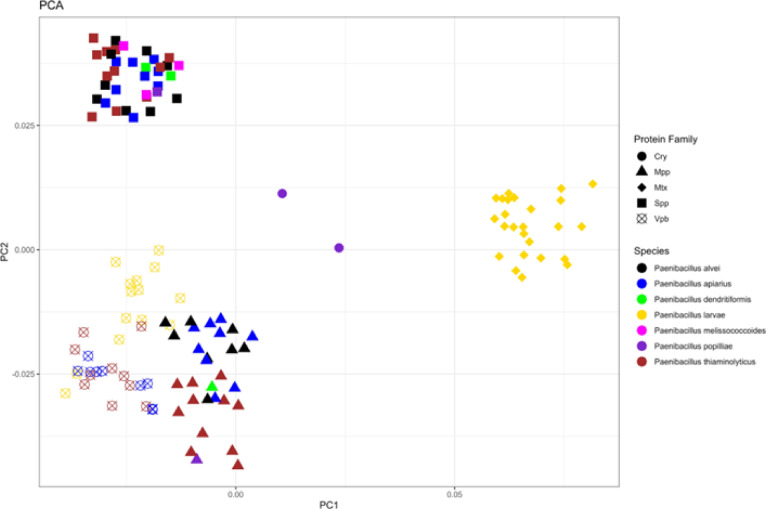
PCA of Putative Insecticidal Toxin Sequences. Amino acid sequences for proteins fulfilling BLAST parameters were converted into frequency tables depicting the position-specific frequency of a given amino acid at a given position in the sequence. These composition tables were then used to create the PCA. Colors of the individual points represent species identity and shapes represent toxin protein family identity.

PCA analysis revealed that BPPs formed three clusters based on their protein family (Fig. 3). The first clustered consisted of Spp toxins from different bacterial species, the second cluster consisted of Mtx toxins from *P. larvae*, and the third cluster consisted of Vpb and Mpp toxins. Since the Spp-type toxins do not form smaller subclusters according to species, we can determine that the Spp-type toxin is highly conserved amongst all members who contain the toxin. *P. apiarius* and *P. alvei* Mpp type toxins appear to be interspaced and cluster together. However, this cluster is separate from the *P. thiaminolyticus* cluster, which appears to spread out further along the space of the graph (Fig. 3). *P. melissococcoides* and *P. dendritiformis* Mpp toxins also seem to be more closely related to the *P. thiaminolyticus* toxins rather than the *P. apiarius* and *P. alvei* group.

**Fig. 4:**
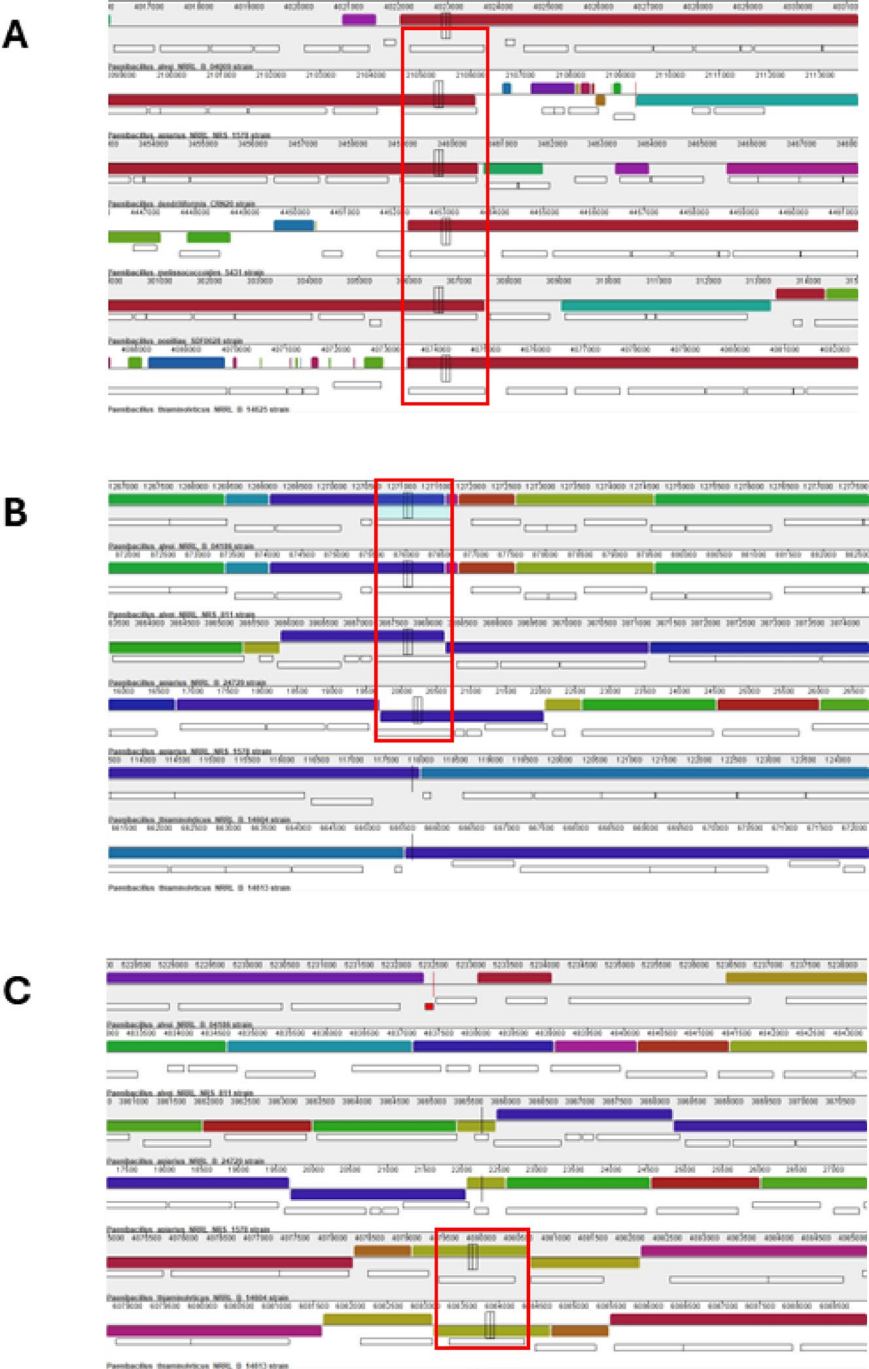
Genomic alignment of toxin clusters. (**A**) Genomic alignment of a subset of Spp-containing strains. The predicted Spp-encoding gene has been highlighted in a red box. (**B**) Genomic alignment of a subset of Mpp containing strains based on the conserved location found in *P. alvei* and *P. apiarius*. Predicted Mpp-encoding genes are highlighted in a red box. (**C**) Genomic alignment of same subset of Mpp-containing strains found in Panel B, but depicting the location conserved between two *P. thaiminolyticus* strains. Predicted Mpp-encoding genes are highlighted in a red box.

**Fig. 5:**
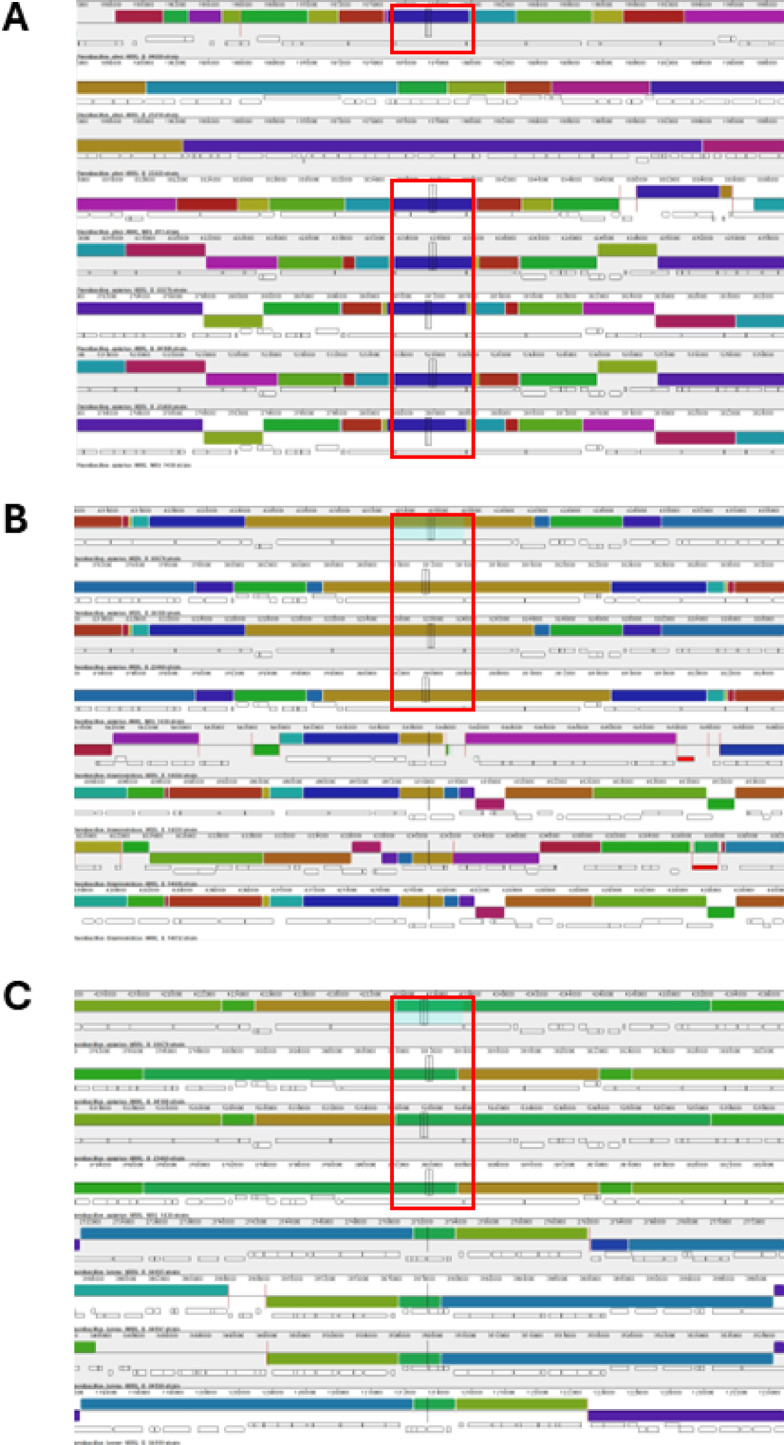
Genomic alignment of Tc Toxin clusters. (**A**) Genomic alignment of *P.apiarius* and *P. alvei* to identify TcABC toxins. (**B**) Genomic alignment of *P. apiarius* and *P. thiaminolyticus* to identify TcABC toxins.(**C**) Genomic alignment of *P. apiarius* and *P. larvae* to identify TcABC toxins. For all panels the gene encoding TcB is highlighted by a red box.

We performed MAUVE alignments of a subset of the toxin-positive strains to identify if the genomic regions were also conserved. We found that the Spp toxin in each selected strain is encoded towards the end of a large metabolic cluster of genes (Fig. 4A). The neighborhood around this metabolic cluster seems to vary significantly between strains. Also, the cluster’s orientation appears to be inverted in several strains, but it is unclear if this is a true inversion or simply an artifact from the WGS coupled with the alignment methodology, considering these alignments were made using genome contigs. While we are confident in the WGS assemblies, long read sequencing should be completed to validate these results on genome organization. For the Mpp type toxins, *P. alvei* and *P. apiarius* strains appear to have similar genomic regions (Fig. 4B). However, the region in the *P. alvei* strains appear to be smaller and lacking some of the additional genes that are present in *P. apiarius* strains. Interestingly, the *P. thiaminolyticus* strains do not align at all with *P. alvei* or *P. apiarius* (Fig. 5B). The *P. thiaminolyticus* Mpp type toxins are in a separate genomic location with a completely different genomic neighborhood and only properly align to themselves (Fig. 4C).

### Entomopathogens contain several ancillary toxin systems found amongst disparate taxa

To ensure a comprehensive search for toxins amongst these genomes, we utilized a HMMER search with approximately 2 million bacterial toxin sequences and searched through each of our genes for suitable hits. We uncovered a plethora of toxin systems that were missed in the initial screening. The most interesting was the presence of a TcABC toxin system found in many strains of *P. apiarius*, *P. alvei*, and *P. thiaminolyticus.* The Tc toxin system functions as a Type VI secretion system, which can deliver a toxin using a syringe-like injection system. Also of interest are P-47 pore-forming proteins often found in clostridial species, and homologs of Plx2-type genes, which function as ADP-ribosyltransferases and are important for *P. larvae* strains that rely on toxin expression for pathogenicity^7,12,13^. Despite being known insecticidal toxins, these ADP-ribosyltransferases were not identified using either the BPPD database or the BtToxin_digger program, highlighting the importance of using multi-faceted analysis when informatically searching through bacterial genomes. The TcABC toxins and Plx2-type toxins appear to exist within the same genomic region and some species share overall genetic structure of the toxin region with *P. apiarius* and *P. alvei* showing the highest level of conservation amongst each other and contain the largest fragments. Of additional interest is the presence of a transposon near the beginning of the cluster site, potentially indicating the historical evidence of horizontal transfer that has occurred to allow this fragment to enter these strains. TcABC toxins often have a hypervariable region on C terminus of TcC which in general is the component that contains the actual toxin function. Based on HMM domain searcher, the C terminus on all the identified TcC proteins appear to be ADP- ribosyltransferases. To have a better understanding of the genomic context of this interesting region containing the Tc toxins, we constructed additional MAUVE alignments to identify conservation of both the Tc toxin system itself and the genomic neighborhood. We found the alignment of *P. apiarius* strains and *P. alvei* strains reveal that only two of the four *P. alvei* strains share the Tc toxin system (Fig. 5A). We looked at a subset of *P. thiaminolyticus* (Fig. 5B) and *P. larvae* strains (Fig. 5C), comparing four sets of genomes to the previously used *P. apiarius strains*. Interestingly, neither the strains selected for *P. thiaminolyticus* or *P. larvae* showed evidence of encoding a TcABC toxin system. Based on these alignments it seems that *P. apiarius* has the greatest representation of Tc toxins, followed by *P. alvei*.

**Fig. 6:**
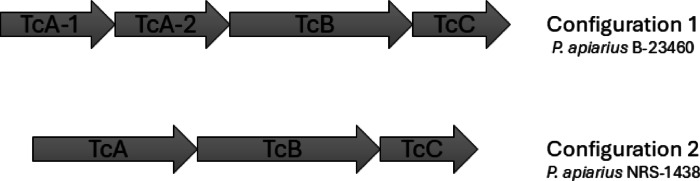
InterProScan Output of Domain Prediction in Putative *P. apiarius* NRS-1438 TcABC Toxin. (**A**) The domains of the predicted TcA protein. (**B**) The domains of the predicted TcB protein. (**C**) The domains of the predicted TcC protein.

We further investigated the TcABC toxins in a subset of *P. apiarius* strains to look for conserved toxin domains that have been identified in known and functional TcABC toxins in other bacteria, such as *Salmonella* and *Yersinia*^47^. We found that these putative TcABC systems contained domains commonly conserved in TcA (Figure S2A), TcB (Figure S2B) and TcC (Figure S2C) in other organisms. indicating that these predicted toxins likely have the domains necessary to be functional. We also found that, even amongst closely related *P. apiarius* strains, the TcABC toxins can exist in different configurations. The system consisted of either a three gene operon, TcABC, or a four gene operon, TcA1A2BC. In the four-gene operon configuration, the TcA gene is not simply duplicated, but rather seem to split the share of domains found in the singular TcA toxin found in the three-gene operon system (Fig. 6).

## Discussion

This study provides evidence for entomopathogenic potential of a known clade of *Paenibacillus* that is evolutionary distinct from *P. larvae*, a devastating pathogen of bees. While the presence of insecticidal toxins in some of these bacterial species is well documented, the pathogenic potential of certain members of the clade such as *P. apiarius*, *P. thiaminolyticus*,* P. dendritiformis*, and *P. melissococcoides* remains unclear. Our results show that this clade contains conserved insecticidal toxins, notably Spp and Mpp like toxins. Theses toxins are structurally and mechanistically distinct from MTX1 toxins found in toxigenic *P. larvae* strains that result in infection of honeybee larvae. Spp toxins are so named due to their homology to the original member of the family, sphaericolysin, originally identified in *Lysinibacillus sphaericus*. Homologs of sphaericolysin are found in diverse bacterial species, including human pathogens such as *Clostridium perfringens*, *Streptococcus pyogenes*, *Listeria monocytogenes* and *B. cereus*. Spp toxins are cytolysins and form pores that disrupt cell membranes by interacting with cholesterol^29^.

Presumably, most bacteria would not produce a toxin unless it serves a distinct purpose required for their lifestyle. Spp like toxins are likely maintained to support the pathogenic lifestyle of the bacterium since they are known to bind to cholesterol in insect or mammalian cells leading to pore formation and cell lysis. Spp like toxins may also be a compelling mechanism that enable these bacteria to compete with protists in the environment by targeting cholesterol that is present in their membrane. However, it is unclear whether these toxins can elicit antifungal or phytotoxic activity by binding to other similar sterols such as ergosterol and phytosterol, respectively. We found the Spp toxins to be highly homologous with percent identity greater than 85% across all *Paenibacillus* species. These findings suggest that. Spp toxins may be a very recent evolutionary acquisition by these strains, such that not enough time has passed for notable drift to occur. Alternatively, it is possible that the genes are actively maintained due to some selective pressure to keep these toxins functional, likely related to an anti-eukaryotic activity, either for competition or for pathogenesis.

This clade of *Paenibacillus* also displays conservation of the Mpp type toxins, but unlike the Spp toxins, the Mpp toxins seem to be more genetically distinct between species, resulting in the *P. alvei*,* P. apiarius* cluster and the *P. thiaminolyticus* cluster. It is unclear if there are functional differences related to these differences or if it is simply the result of genetic drift. Since the Mpp toxins appear to exist in completely different genomic areas, it seems likely that *P. alvei* and *P. apiarius* acquired their Mpp toxins through a shared ancestor, while *P. thiaminolyticus* may have acquired it from a different source. Mpp toxins are MTX2 like toxins, often referred to as mosquitocidal toxins due to their well-documented activity against mosquitoes. Mpp toxins are thought to act by forming pores in the membrane of the host cells, but there is no evidence to suggest specific targets. It is possible that these differences are related to different host specificities or differences in toxicity, but additional testing is needed to confirm this speculation.

A very surprising finding from this study was the presence of TcABC toxin systems, normally found in Gram-negative bacteria. These systems have high homology to those of *Photorhabadus luminescens*, *Salmonella enterica*, and *Yersinia entemophaga*^47–49^. The Tc toxin system functions as a Type VI secretion system, which form a syringe-like injection system that can bind to and penetrate target membranes to deliver a toxin^47,49,50^. Few studies have investigated expression of a Tc toxin system in a few strains of *B. thuringiensis*^48^. However, there is limited information on the functionality of Tc systems in Gram-positive bacteria, especially their insecticidal potential. TcABC toxin system in *P. luminesces* has been shown to interact with a Vsg specific receptor in Drosophila cells^51^. The Vsg receptor is conserved across a variety of insect orders, indicating that TcABC toxin specificity could either be broad or narrow range depending on the receptor target. In addition to the Tc toxin system, we also identified several ADP- ribosyltransferases, which function by targeting Rho GTPases in eukaryotic cells^52^. ADP- ribosyltransferases are known to be major contributors of pathogenicity in *P. larvae*^12^, but have not been well studied in other *Paenibacillus* species. We have found several potential pathogenicity islands that seem to have high insecticidal potential among these *Paenibacillus* strains. These toxins could play an important role in increasing the insect toxicity and host specificity of these strains. Further studies are needed to determine the functional role of these toxin systems in these bacteria.

Since the *Paenibacillus* strains examined in this study were identified on dead honeybee larvae, a naïve assumption would be that honeybees are a possible target for these pathogens. However, if this was the case, many of these *Paenibacillus* pathogens should often be detected in live bees as is the case for *P. larvae*. While species misidentification could hinder the ability to link a pathogen to a specific disease, it appears likely that honeybees are not the prime target for the other *Paenibacillus* in this putative entomopathogenic clade. It could be these are opportunistic pathogens that are passively vectored into hives and occasionally successful kill larvae that are abiotically or biotically stressed. Our results show that the two clades are taxonomically distinct. The *Paenibacillus* genus has become very large containing more than 411 species (https://lpsn.dsmz.de/genus/paenibacillus). Using the Genome Taxonomy Database that uses relative evolutionary divergence to delineate taxa, the *Paenbacillus* genus should be split into at least 26 separate genera^53^. In addition, it would place the entomopathogenic clade described here in a genus separate from *Paenibacillus sensu stricto* while *P. larvae* would be placed in a different bacterial family outside of Paenibacilleaceae. We hypothesize that these bacteria are likely targeting other insects and further studies are needed to determine the range of insects that can be affected by these bacteria. These findings further suggest that strains in the putative entomopathogenic clade are evolutionarily distant from the known bee pathogen, *P. larvae* and they evolved separately. If the taxonomy was updated as proposed, these clades would exist in different bacterial families.

Currently, most research on the use of bacteria or their products to control insect pests primarily focus on *B. thuringiensis* strains. Our results show that putative entomopathogenic clade of *Paenibacillus* contain toxins that could be investigated further for application as biopesticides. We have identified several classic insecticidal toxins systems within this clade, including Spp, Mpp, and Vpa/b binary toxin systems. We have also identified several systems that may not be immediately associated with insecticidal potential, such as TcABC toxins and several ADP- ribosyltransferases toxins. This clade should be investigated further for their potential as biocontrol agents for insect control. With such varied toxin systems, it is possible that these bacteria have a host range which could be useful for application in crop protection. The specificity of some of the toxins, such as the receptor binding of the TcABC toxin systems is well known and could be leveraged to provide insecticidal activity against pests of economic significance while preserving and protecting vulnerable honeybee populations.

## Electronic supplementary material

Below is the link to the electronic supplementary material.

Supplementary Material 1

Supplementary Material 2

## Data Availability

All data generated during this study is available under GenBank Bioproject PRJNA805124.
