# Supplementary material for: Characterization of toxin systems of Paenibacillus strains isolated from honeybees
Source: Sci Rep. 2025 Aug 26;15:31346. doi: 10.1038/s41598-025-12956-x (PMC12381145; doi:10.1038/s41598-025-12956-x)
Supplement: Supplementary file 1 — Supplementary Material 1 [file 41598_2025_12956_MOESM1_ESM.pdf]

SUPPLEMENTAL FIGURES

for

Characterization of toxin systems of Paenibacillus strains isolated from honeybees

Anthony Pannullo, Ephantus J. Muturi and Christopher A. Dunlap[*]

Crop Bioprotection Research Unit, National Center for Agricultural Utilization Research, Agricultural Research Service, United States Department of Agriculture, Peoria, IL, USA

*Address correspondence to Christopher A. Dunlap, christopher.dunlap@usda.gov

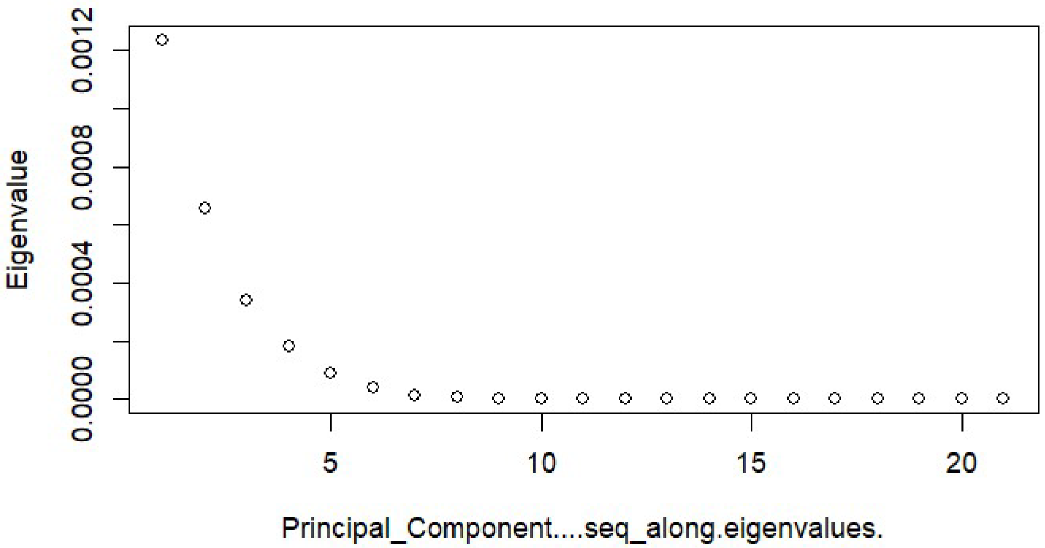

Figure S1: Scree Plot displaying the amount of variance explained by each principal component

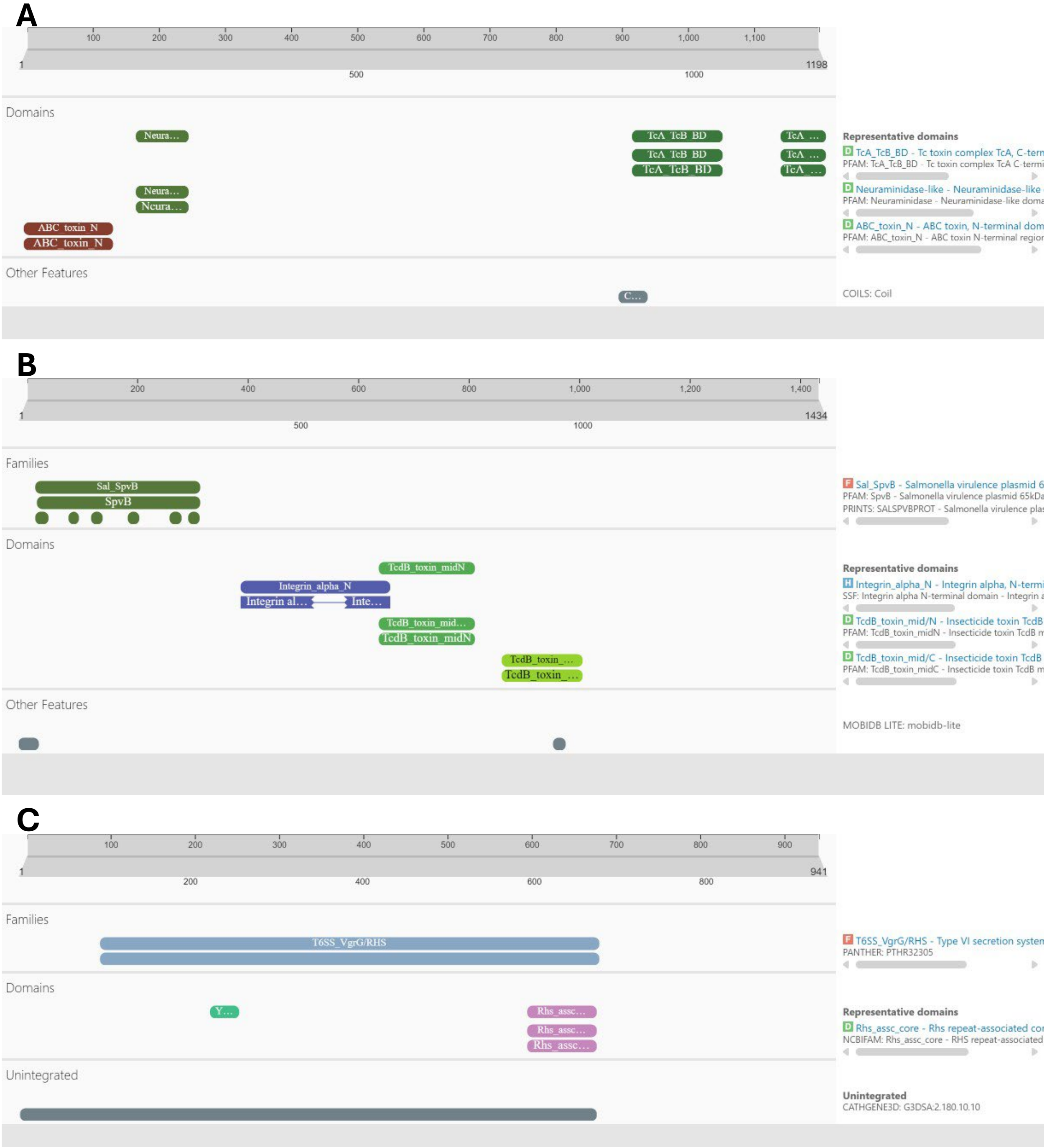

Figure S2: (A) InterProScan Output of Domain Prediction in Putative *P. apiarius* NRS-1438 TcABC Toxin. (A) The domains of the predicted TcA protein. (B) The domains of the predicted TcB protein. (C) The domains of the predicted TcC protein.
